# Translating laboratory quality management system into practice: strategic opportunities and challenges for ISO 15189:2022 implementation in Nigeria's healthcare system

**DOI:** 10.3389/frhs.2026.1850576

**Published:** 2026-07-31

**Authors:** Bruno Basil, Ngozi Ijeoma Okoro, Izuchukwu Nnachi Mba, Blessing Kenechi Myke-Mbata

**Affiliations:** 1 International Institute of Pathology and Forensic Science Research, David Umahi Federal University of Health Sciences, Uburu, Nigeria; 2 Department of Chemical Pathology, Enugu State University of Science and Technology, Enugu, Nigeria; 3 Department of Chemical Pathology, Nile University of Nigeria, Abuja, Nigeria; 4 Department of Chemical Pathology, Benue State University, Makurdi, Nigeria

**Keywords:** clinical laboratories, ISO 15189:2022, laboratory accreditation, laboratory quality management system (LQMS), Nigeria, quality improvement

## Abstract

**Introduction:**

Laboratory Quality Management Systems (LQMS) are fundamental to reliable diagnostic services and improved patient outcomes. Although Nigeria has made progress through quality improvement initiatives, implementation of internationally recognized standards remains limited. This review critically examines the relevance of ISO 15189:2022 for strengthening laboratory quality systems in Nigeria by evaluating its major revisions, current implementation status, challenges, opportunities, and evidence-based strategies for national adoption.

**Methods:**

A structured narrative review was conducted using evidence published between January 2010 and July 2026. Electronic databases (PubMed, PubMed Central, Web of Science, and Google Scholar) and institutional sources (WHO-AFRO, ASLM, MLSCN, NiNAS, and ILAC) were systematically searched. Evidence selection followed a PRISMA-informed process, while reporting adhered to the SANRA guideline. Of 379 records identified, 55 met the inclusion criteria, with 49 synthesized thematically and six retained for foundational and conceptual support.

**Results:**

The evidence showed that LQMS implementation in Nigeria remains heterogeneous, with stronger performance in donor-supported reference laboratories than in many public facilities. SLMTA and SLIPTA programmes have improved laboratory audit performance, including in Lagos State, yet fewer than 30 of Nigeria's over 5,000 clinical laboratories are currently ISO 15189 accredited. Major barriers include fragmented governance, inadequate financing, weak infrastructure, workforce shortages, limited digital integration, and poor quality culture. ISO 15189:2022 introduces patient-centred, risk-based quality management, integrated point-of-care testing, and flexible documentation, creating opportunities to modernize laboratory practice through strengthened governance, digital transformation, workforce development, and sustained investment.

**Conclusion:**

ISO 15189:2022 provides a practical framework for improving laboratory quality and technical competence in Nigeria. Successful implementation will require coordinated regulatory reforms, expanded stepwise quality improvement programmes, sustained financing, workforce capacity building, and digital innovation to strengthen diagnostic services and support resilient health systems.

## Graphical Abstract

**Figure:**
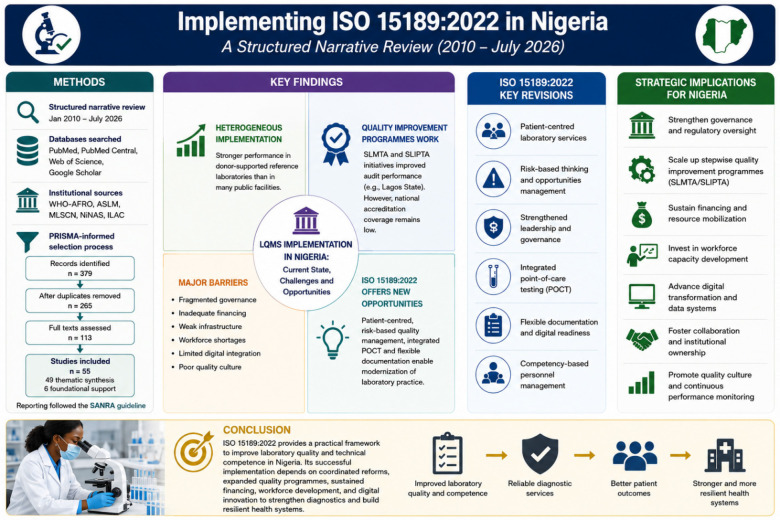

## 1 Introduction

Reliable and accurate diagnostics are the cornerstone of effective healthcare systems. They influence over 70% of clinical decision-making, from disease screening and diagnosis to treatment monitoring and prognosis (1–3). However, in Nigeria, the quality of diagnostic services remains substandard, often contributing to misdiagnoses, delayed treatment initiation, poor patient outcomes, and increased healthcare costs, issues that are largely attributable to weak laboratory systems that lack robust quality assurance frameworks (4).

Nigeria's laboratory network is expansive, comprising more than 5,000 public, private, and research-based clinical laboratories. These laboratories play a pivotal role in the diagnosis and management of high-burden diseases such as HIV, tuberculosis, malaria, non-communicable diseases, and more recently, emerging and re-emerging infectious diseases (5). Despite this strategic importance, the laboratory sector continues to face systemic challenges including fragmented governance, inadequate financing, workforce shortages exacerbated by health professional migration, aging infrastructure, obsolete equipment, limited digital integration, and inconsistent adherence to quality standards (4, 6, 7). These limitations severely constrain the delivery of timely, reliable, and patient-centered diagnostic services.

Although national policies have increasingly emphasized laboratory strengthening, the practical implementation of a Laboratory Quality Management System (LQMS) remains suboptimal across most Nigerian laboratories. An LQMS is a comprehensive, process-oriented framework designed to manage all components of laboratory operations to ensure quality and competence (8, 9). The World Health Organization (WHO) advocates for a model based on 12 Quality System Essentials (QSEs), which include key domains such as personnel, equipment, process control, information management, and continual improvement. However, translating this structured approach into practice is hindered by inadequate infrastructure, inconsistent standards, and weak regulatory enforcement (6, 9).

Against this backdrop, the ISO 15189:2022 standard offers an updated and integrated model for improving laboratory quality and competence. It was originally developed in the early 2000s to address the specific needs of Clinical laboratories not fully covered by ISO 9001, drawing on the technical competence principles of ISO/IEC 17025, and has undergone several revisions in 2003, 2007, and 2012 to reflect changes in laboratory science and healthcare demands (10, 11). The 2022 revision marks a significant evolution, emphasizing patient-focused risk management, integrating requirements for Point-of-Care Testing (POCT), and aligning structurally with ISO/IEC 17025:2017 to facilitate accreditation and global harmonization (11–13). Following the completion of the global transition from ISO 15189:2012 in December 2025, ISO 15189:2022 now serves as the internationally accepted benchmark for quality and competence in medical laboratories. This transition has heightened the need for laboratories, particularly in resource-constrained settings such as Nigeria, to strengthen institutional capacity, align quality management systems with the revised requirements, and sustain accreditation readiness (14). Although previous studies have examined laboratory quality management and earlier ISO 15189 frameworks, evidence evaluating Nigeria's preparedness following the December 2025 global transition to ISO 15189:2022 remains limited. This review provides an updated synthesis of implementation progress, challenges, and strategic priorities.

In this study, we aimed to critically examine the relevance and applicability of ISO 15189:2022 within the Nigerian context. Specifically, we analyzed the major updates in ISO 15189:2022, assessed the current state of LQMS implementation in Nigeria, identified the key challenges and enabling factors affecting adoption, and proposed evidence-based strategies to support effective national uptake of the standard. By promoting structured quality management and accreditation readiness, ISO 15189:2022 implementation has the potential to strengthen Nigeria's diagnostic ecosystem. It aligns with broader national and global health goals, including the achievement of Universal Health Coverage (UHC) and the reinforcement of public health surveillance systems for outbreak preparedness and response (14, 15). As such, the strategic adoption of ISO 15189:2022 is not merely a technical endeavour, but a critical step toward sustainable, high-quality healthcare delivery in Nigeria.

## 2 Methods

### 2.1 Study design

This study employed a structured narrative review to examine the implementation of Laboratory Quality Management Systems (LQMS) and ISO 15189:2022 in Nigeria. Evidence was synthesized from peer-reviewed literature, regulatory publications, accreditation guidelines, policy documents, and implementation reports to evaluate the current state of laboratory quality management, identify implementation challenges, and propose strategies for strengthening laboratory systems. A systems perspective was adopted to integrate evidence on governance, regulatory oversight, accreditation, workforce development, financing, infrastructure, quality improvement programmes, and implementation experiences from Nigeria and comparable sub-Saharan African countries.

### 2.2 Conceptual framework

**Figure 1:**
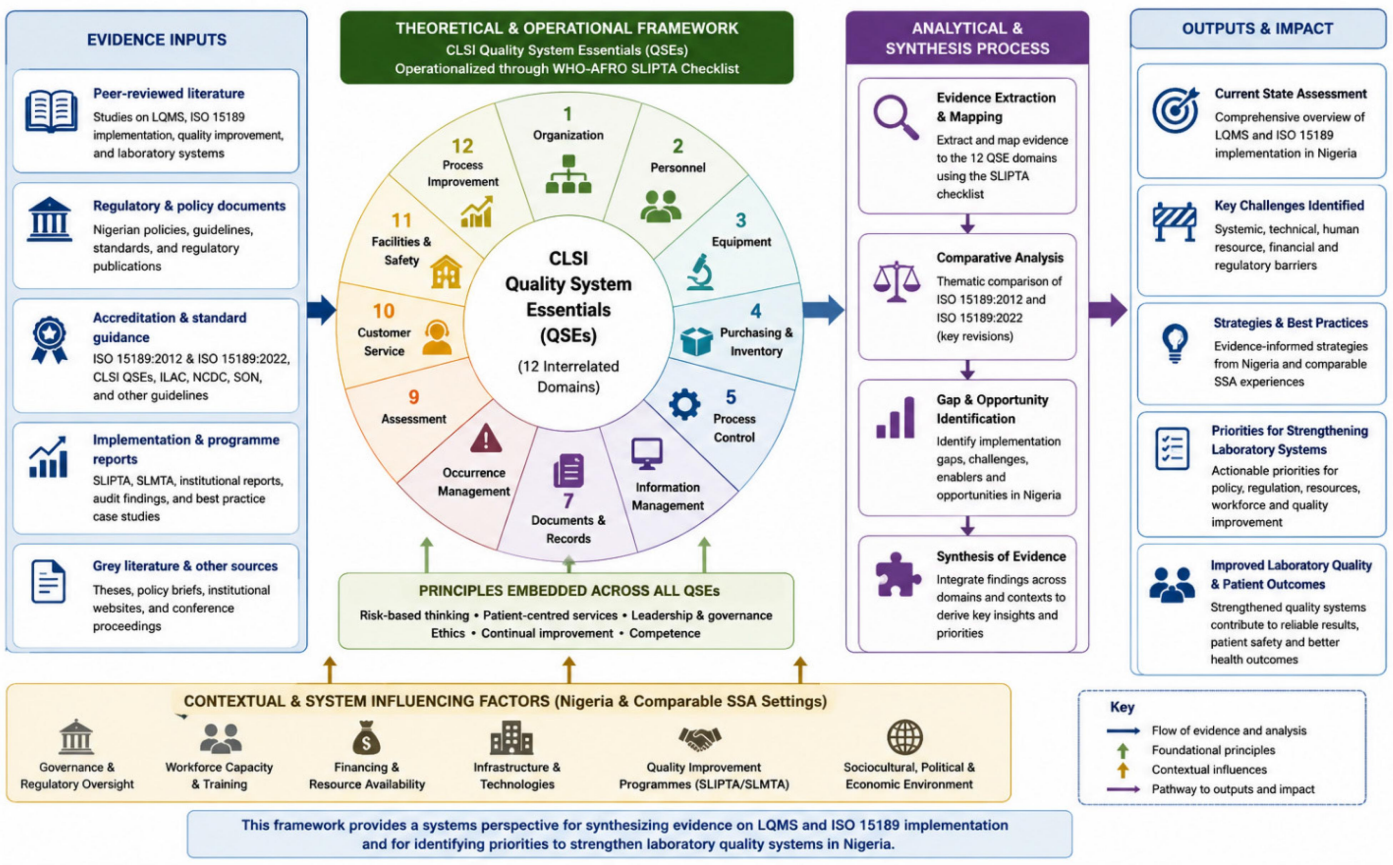
Conceptual framework illustrating the evidence synthesis approach used in this narrative review. The framework is based on the CLSI Quality System Essentials (QSEs), operationalized through the WHO-AFRO SLIPTA framework, and applied to examine the implementation of Laboratory Quality Management Systems (LQMS) and ISO 15189:2022 in Nigeria.

The review was guided by the Clinical and Laboratory Standards Institute (CLSI) Quality System Essentials (QSEs) framework, operationalized through the World Health Organization Regional Office for Africa (WHO-AFRO) Stepwise Laboratory Quality Improvement Process Towards Accreditation (SLIPTA) checklist (9, 16, 17). The twelve QSEs served as the primary framework for organizing and interpreting the evidence. A thematic comparison of ISO 15189:2012 and ISO 15189:2022 was also undertaken to examine major revisions related to risk-based thinking, patient-centred laboratory services, leadership and governance, point-of-care testing, documentation, and competency-based quality management. Evidence was subsequently mapped to these domains to identify implementation gaps, opportunities, and priorities for strengthening laboratory quality systems in Nigeria (Figure 1).

### 2.3 Information sources, search strategy and eligibility criteria

A comprehensive literature search was conducted to identify evidence published between January 2010 and July 2026, encompassing the expansion of laboratory quality improvement initiatives across Africa and the global transition to ISO 15189:2022 following the December 2025 implementation deadline. Electronic searches were performed in PubMed, PubMed Central (PMC), Google Scholar, and ResearchGate, while grey literature, accreditation manuals, regulatory publications, and policy documents were retrieved from the official websites of the World Health Organization Regional Office for Africa (WHO-AFRO), African Society for Laboratory Medicine (ASLM), Medical Laboratory Science Council of Nigeria (MLSCN), Nigeria National Accreditation System (NiNAS), and the International Laboratory Accreditation Cooperation (ILAC). To maximize retrieval of relevant evidence, supplementary hand-searching of reference lists from eligible articles, key review papers, guideline documents, and official reports was also undertaken, together with forward citation tracking of seminal publications and targeted searches for recently published studies.

Search terms were organized into four thematic clusters:

- **Cluster 1 (ISO Standards and Laboratory Quality Management):** “ISO 15189”, “ISO 15189:2022”, “ISO 15189:2012”, “transition”, “gap analysis”, “risk-based thinking”, “patient safety”, and “point-of-care testing (POCT)”.
- **Cluster 2 (Laboratory Quality Implementation):** “Quality Management System”, “LQMS”, “SLMTA”, “SLIPTA”, “continuous quality improvement”, “Nigeria”, “Lagos State”, “sub-Saharan Africa”, and “resource-constrained settings”.
- **Cluster 3 (Regulatory and Accreditation Systems):** “Medical Laboratory Science Council of Nigeria”, “MLSCN”, “Nigeria National Accreditation System”, “NiNAS”, “accreditation”, “licensure”, “competence”, and “regulatory guidelines”.
- **Cluster 4 (Health System Challenges):** “brain drain”, “Japa”, “workforce attrition”, “financial cost”, “supply chain”, “reagent stock-out”, “clinical laboratory”, and “public hospital”.

**Figure 2:**
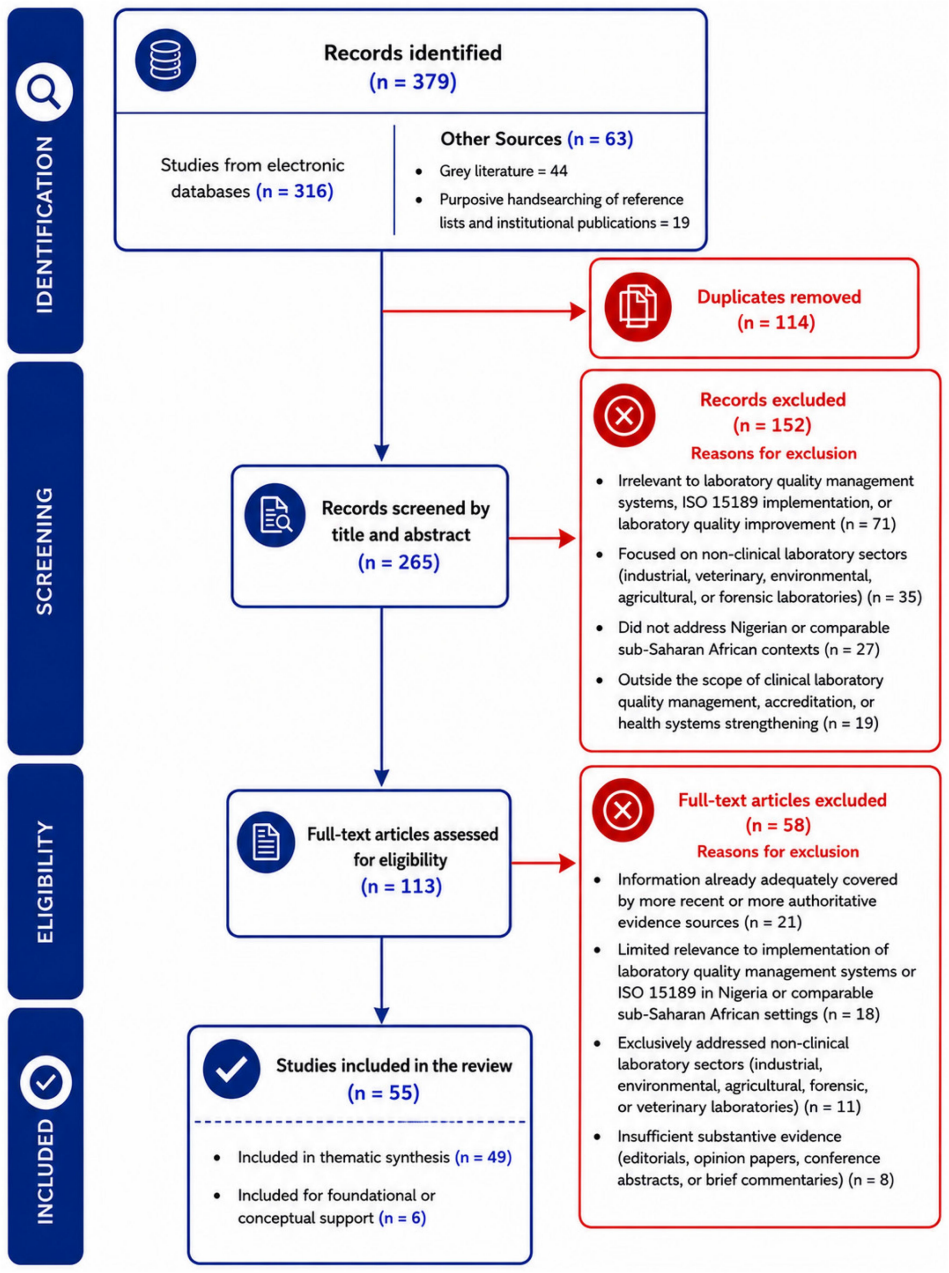
PRISMA-informed flow diagram illustrating the identification, screening, eligibility assessment, and inclusion of evidence sources informing this narrative review.

Eligible evidence included original research articles, implementation studies, systematic and narrative reviews, quality improvement reports, accreditation assessments, regulatory publications, statutory documents, and policy frameworks addressing laboratory quality management, ISO 15189 implementation, laboratory accreditation, or health system strengthening in Nigeria and comparable sub-Saharan African settings. Publications focusing exclusively on veterinary, agricultural, industrial, or environmental laboratories, conference abstracts, editorials, opinion articles without substantive evidence, dissertations, and studies lacking relevance to clinical laboratory quality management were excluded. Only English-language publications were considered. The review was conducted and reported in accordance with the Scale for the Assessment of Narrative Review Articles (SANRA) to enhance methodological transparency and reporting quality (18), while study identification, screening, and selection followed a PRISMA-informed approach (Figure 2).

### 2.4 Evidence selection, data extraction and synthesis

Study selection followed a PRISMA-informed process adapted for narrative reviews (Figure 2). A total of 379 records were identified through electronic database searches, grey literature, and supplementary hand searching of reference lists and institutional publications. After the removal of 114 duplicates, 265 unique records underwent title and abstract screening. Following full-text eligibility assessment of 113 articles, 55 sources met the inclusion criteria. Of these, 49 were included in the thematic synthesis, while 6 additional authoritative publications were retained to provide foundational and conceptual support for the review.

Data were extracted using a standardized framework capturing study characteristics, laboratory setting, quality management interventions, accreditation status, implementation strategies, barriers, facilitators, outcomes, and policy implications. Evidence was synthesized narratively using thematic analysis, with findings organized into the major themes of the review. Quantitative findings reported in the included studies were summarized descriptively. Comparative evidence from Nigeria and other sub-Saharan African countries was integrated to identify recurring implementation patterns and transferable lessons. Figures and summary tables were developed through thematic synthesis of the included evidence.

## 3 Results

### 3.1 Current state of LQMS in Nigeria

Nigeria's clinical laboratory network comprises over 5,000 public, private, faith-based, military, and research laboratories that support disease diagnosis, treatment monitoring, and public health surveillance for a population exceeding 220 million (19). Although the country has established regulatory and accreditation structures through the Medical Laboratory Science Council of Nigeria (MLSCN) and the Nigeria National Accreditation System (NiNAS), implementation of Laboratory Quality Management Systems (LQMS) remains heterogeneous across laboratory tiers and ownership categories. Existing evidence indicates that laboratory quality is generally stronger in donor-supported reference laboratories and a small number of private facilities, while many public secondary and primary laboratories continue to operate with limited quality management capacity and inconsistent compliance with international standards (4, 7, 20, 21). Regulatory oversight remains constrained by overlapping institutional mandates and fragmented governance, which continue to impede uniform implementation of quality standards (4, 7, 22).

National efforts to strengthen laboratory quality have largely been driven by the WHO-AFRO Strengthening Laboratory Management Toward Accreditation (SLMTA) programme and the Stepwise Laboratory Quality Improvement Process Towards Accreditation (SLIPTA), which provide structured frameworks for implementing the 12 Quality System Essentials (QSEs) (23, 24). These initiatives have improved quality management capacity in participating laboratories; however, national coverage remains limited. Despite recent growth in accreditation, ISO 15189-certified laboratories still constitute only a small proportion of Nigeria's diagnostic network, reflecting persistent gaps in infrastructure, financing, workforce capacity, governance, and institutional commitment (4, 25).

Available evidence nevertheless demonstrates that measurable improvements are achievable when quality improvement programmes are systematically implemented. Government-led interventions in Lagos State showed substantial improvements in SLIPTA performance following structured training, mentorship, supportive supervision, and resource mobilization, with 60% of participating laboratories attaining 3-star status and 20% achieving 2-star status after one year (7). Similarly, several Nigerian public, military, research, and private laboratories have successfully attained and maintained ISO 15189 accreditation, demonstrating that sustained leadership commitment, investment in quality systems, and continuous mentorship can overcome many implementation barriers. However, inadequate infrastructure, unreliable electricity supply, obsolete equipment, inadequate financing, and persistent workforce shortages continue to constrain nationwide implementation (4, 23). Collectively, these experiences suggest that the principal challenge in Nigeria is not the feasibility of accreditation, but scaling successful models across the broader laboratory network. Table 1 summarizes the current evidence on laboratory quality management implementation in Nigeria.

**Table 1:** Current status of laboratory quality management systems (LQMS) implementation in Nigeria.

Domain	Current evidence	Implications
Laboratory network	>5,000 clinical laboratories across public, private, research, military and faith-based sectors	Large diagnostic capacity but marked heterogeneity in quality systems
Regulatory framework	MLSCN regulates medical laboratory practice; NiNAS provides internationally recognized accreditation	Regulatory structures exist but implementation remains inconsistent
Quality improvement programmes	WHO-AFRO SLMTA and SLIPTA adopted in selected laboratories	Demonstrated improvements but limited national coverage
Accreditation status	ISO 15189-accredited laboratories remain a small fraction of Nigeria's laboratory network despite gradual increases in recent years	National accreditation uptake remains low
Donor-supported programmes	Most mature QMS implementation occurs within HIV, TB and public health programmes	Quality improvements are often programme-specific rather than facility-wide
Demonstrated success stories	NIMR, 661 Nigerian Air Force Hospital Laboratory, SYNLAB Nigeria, Cerba Lancet Africa, Afriglobal Medicare Ltd., Link Medical Diagnostic and Research Laboratories, Agape Laboratory Services, and Lagos State public laboratory programme	Demonstrates feasibility of ISO 15,189 implementation under sustained institutional support
Lagos State QMS intervention (2022–2023)	Following structured mentorship and capacity building, 60% of participating laboratories attained 3-star SLIPTA status and 20% achieved 2-star status	Government-led quality improvement programmes can rapidly strengthen LQMS in resource-constrained settings
Major implementation gaps	Inadequate infrastructure, unreliable power supply, equipment maintenance challenges, workforce shortages, fragmented governance and limited financing	Continue to constrain nationwide implementation and sustainability of LQMS

The global transition to ISO 15189:2022 has shifted the focus of laboratory quality management beyond traditional quality assurance toward integrated, patient-centred, and risk-based quality systems (13). Following the December 2025 transition deadline, accredited medical laboratories are undergoing reassessment and progressive implementation of the revised standard. As this transition remains ongoing, the full extent of implementation and accreditation outcomes continues to evolve, with country-specific evidence still emerging (12, 13). Compared with the 2012 edition, the revised standard introduces several changes with important implications for laboratories in resource-limited settings such as Nigeria. While these revisions provide opportunities to modernize laboratory practice, they also increase the technical and organizational requirements for successful implementation (Table 2) (10–13).

**Table 2:** Key changes in ISO 15189:2022 compared with ISO 15189:2012 and their implications for Nigerian laboratories.

Change area	ISO 15189:2012	ISO 15189:2022	Implications for Nigerian laboratories
Risk management	Primarily corrective and preventive actions (CAPA)	Proactive, patient-centred risk management integrated throughout the QMS	Requires a shift from reactive quality assurance to continuous risk-based management
Point-of-care testing (POCT)	Covered separately under ISO 22870	Incorporated within ISO 15189 (Annex A)	Strengthens oversight of POCT but presents implementation challenges, particularly in primary healthcare settings
Documentation	Mandatory quality manual	Flexible documentation aligned with ISO/IEC 17025	Allows adaptable documentation systems but requires greater QMS maturity
Patient focus	General user requirements	Greater emphasis on patient welfare, clinical consultation and user satisfaction	Encourages stronger laboratory-clinician collaboration and patient-centred service delivery
Terminology and definitions	Limited standardized definitions	Expanded and clarified terminology (e.g., impartiality, commutability, patient)	Promotes consistent interpretation but necessitates additional staff training and competency development

### 3.2 Strategic opportunities for ISO 15189:2022 implementation in Nigeria

The transition to ISO 15189:2022 presents an opportunity to strengthen Nigeria's laboratory quality management system beyond conventional quality assurance by promoting patient-centred, risk-based, and digitally enabled laboratory practice. The revised standard aligns with ongoing national efforts to improve diagnostic quality and provides a framework through which laboratory strengthening initiatives can be integrated into broader health system reforms.

A major opportunity lies in leveraging existing national and international quality improvement programmes. The WHO-AFRO Strengthening Laboratory Management Toward Accreditation (SLMTA) and Stepwise Laboratory Quality Improvement Process Towards Accreditation (SLIPTA) initiatives have demonstrated that structured training, mentorship, and stepwise quality improvement can substantially enhance laboratory performance in resource-constrained settings (24, 25). Similarly, the Nigerian National Health Laboratory Accreditation Programme and the expanding role of regulatory bodies provide institutional platforms for scaling accreditation and sustaining quality improvement across laboratory tiers (21).

Integration of ISO 15189:2022 into existing health programmes offers another strategic advantage. Embedding laboratory quality requirements within initiatives coordinated by the Federal Ministry of Health, National Health Insurance Authority (NHIA), Nigeria Centre for Disease Control and Prevention (NCDC), and disease-specific programmes could strengthen diagnostic reliability while linking quality performance with financing, surveillance, and service delivery (3, 22). Such alignment would facilitate the transition from donor-driven quality initiatives to nationally owned laboratory systems.

The revised standard also creates opportunities to accelerate digital transformation within laboratory services. Increased adoption of Laboratory Information Systems (LIS), electronic quality management systems, and digital audit tools can improve traceability, documentation, quality indicator monitoring, and data integration, while supporting the risk-based approach required under ISO 15189:2022 (26). Likewise, strengthening LIS infrastructure would enhance real-time reporting, audit readiness, and integration with national health information systems for improved planning and accountability (27). Furthermore, the incorporation of point-of-care testing (POCT) within the revised standard provides an opportunity to extend standardized quality management to decentralized testing sites, particularly within primary healthcare.

Workforce strengthening remains central to successful implementation. Expanding competency-based training, mentorship, internal auditing capacity, and continuous professional development would equip laboratory personnel with the skills required to implement the revised standard while fostering a sustainable culture of quality (7, 28). Evidence from laboratory quality improvement programmes consistently demonstrates that staff empowerment and continuous professional development are fundamental to successful institutionalization of quality management systems (29). These efforts could be complemented by public-private partnerships that support infrastructure development, technology transfer, mentorship, and sustainable financing, thereby reducing resource constraints that currently limit accreditation (22, 30).

Overall, available evidence suggests that Nigeria possesses several enabling factors for successful implementation of ISO 15189:2022, although these must be matched by sustained political commitment, regulatory coordination, and long-term investment. The principal strengths (7, 9, 21, 23, 31–33), opportunities (3, 10–12, 22–25, 34), weaknesses (23, 25, 31, 35, 36), and threats (4, 6, 20, 22, 37, 38) influencing implementation are summarized in Table 3.

**Table 3:** SWOT analysis of ISO 15189:2022 implementation in Nigeria.

Strengths (Internal, Positive)	Weaknesses (Internal, Negative)
Localized examples of successful implementation such as NIMR and Lagos State's SLIPTA-backed program show feasibility with political commitment and mentorship.	Critically low accreditation rate: fewer than 30 out of >5,000 laboratories are ISO-accredited, reflecting minimal national uptake.
Existence of a national accreditation body, NiNAS, enabling domestic accreditation and reducing reliance on foreign systems.	Widespread systemic non-conformities during audits: poor documentation, internal audits, occurrence management, and corrective actions persist across facilities
Professional regulation through a unified regulatory body provides a formal structure for quality oversight.	Cultural resistance to QMS practices: documentation and internal audits are viewed as unnecessary burdens, undermining adherence to SOPs.
Nigeria's young and adaptable laboratory workforce can be rapidly upskilled through targeted QMS training.	Massive health workforce brain drain: a large number of health workers including laboratory professionals have migrated abroad in recent years, depleting capacity.

Opportunities (External, Positive)	Threats (External, Negative)
WHO's SLMTA and SLIPTA programs offer locally adaptable, stepwise implementation tools already proven in Nigeria and other LMICs.	Chronic underfunding of the health sector leads to poor infrastructure, unreliable electricity, and inadequate logistics for QMS implementation.
PPPs can help bridge gaps in infrastructure, mentorship, and technology by leveraging private investment and expertise.	Regulatory fragmentation and overlapping mandates between MLSCN, NAFDAC, MDCN create conflicting policies and jurisdictional ambiguity.
Release of ISO 15189:2022 emphasizes digitalization, POCT integration, and risk-based thinking, acting as a modernization catalyst.	Inflation, insecurity, and poor working conditions exacerbate workforce attrition, limiting sustained QMS implementation.
Federal health reform agenda prioritizes diagnostics and accreditation, creating alignment opportunities for ISO integration.	Heavy dependence on imported reagents and equipment makes QMS operations vulnerable to foreign exchange volatility and global supply chain disruptions.

### 3.3 Challenges in implementing ISO 15189:2022 in Nigeria

Despite the strategic advantages of ISO 15189:2022, its implementation in Nigeria remains constrained by interrelated regulatory, financial, infrastructural, technical, and workforce challenges that collectively impede the establishment of sustainable laboratory quality management systems. Regulatory and governance challenges remain a major barrier to implementation. Oversight of medical laboratories is shared among the Medical Laboratory Science Council of Nigeria (MLSCN), Medical and Dental Council of Nigeria (MDCN), National Agency for Food and Drug Administration and Control (NAFDAC), and state ministries of health. These overlapping mandates create inconsistencies in policy implementation, particularly in the regulation of *in vitro* diagnostics (IVDs), resulting in fragmented governance and variable enforcement of quality standards (32). This regulatory disconnect represents a critical systemic bottleneck; unlike several successful implementation models reported in Ghana, Kenya, and Malawi, where nationally coordinated quality improvement and accreditation programmes supported more consistent implementation, regulatory fragmentation remains a distinctive constraint limiting scale-up in Nigeria by fracturing institutional accountability.

Resource limitations continue to undermine quality improvement efforts. Chronic underinvestment in laboratory infrastructure, unreliable electricity, inadequate equipment maintenance, weak supply chains, and frequent shortages of reagents and consumables compromise the consistency of laboratory operations and the sustainability of quality systems (4, 39). These constraints are further compounded by the substantial financial costs associated with achieving and maintaining ISO 15189 accreditation, making accreditation unattainable for many laboratories without external support.

Human resource and organizational challenges also remain pervasive. The ongoing migration of trained laboratory professionals has significantly reduced the availability of experienced personnel required to establish and sustain quality management systems (35, 36). Available evidence further indicates considerable gaps in competency and training on ISO 15189:2022, particularly among frontline technical personnel, limiting effective implementation of quality procedures. Resistance to documentation, internal audits, occurrence reporting, and adherence to standard operating procedures also persists in many laboratories, reflecting a weak institutional culture of quality and continuous improvement (5, 39).

Operational deficiencies further hinder implementation. Quality management remains heavily dependent on donor-supported vertical programmes, resulting in fragmented implementation across laboratory units rather than institution-wide quality systems (7, 40). This structural isolation exposes a core weakness in reliance on donor funding, where narrow, disease-specific clinical compliance fails to translate into sustainable, facility-wide quality architecture. In addition, many laboratories inadequately monitor quality indicators such as turnaround time, error rates, customer satisfaction, and corrective actions, limiting the use of quality data for continuous improvement (34).

Audit findings consistently demonstrate recurring weaknesses in documentation, internal audits, occurrence management, quality indicator monitoring, corrective and preventive actions, equipment management, and process validation. These persistent non-conformities continue to limit progression through the SLIPTA framework and ultimately delay attainment of ISO 15189 accreditation (15, 41, 42).

Overall, the evidence indicates that the challenges facing ISO 15189:2022 implementation extend beyond individual laboratories and reflect broader systemic weaknesses within Nigeria's health system. Table 4 summarizes the principal implementation challenges and their implications for laboratory quality improvement.

**Table 4:** Major challenges affecting implementation of ISO 15189:2022 in Nigeria.

Challenge domain	Key evidence	Implications for implementation
Regulatory and governance	Overlapping mandates among MLSCN, MDCN, NAFDAC and state authorities; inconsistent regulation of IVDs (22, 30)	Fragmented oversight, inconsistent policy implementation and weak enforcement of quality standards
Infrastructure and equipment	Inadequate infrastructure, unreliable electricity, poor equipment maintenance and calibration (22, 30)	Equipment downtime, compromised test quality and interrupted laboratory services
Consumables and supply chain	Frequent reagent stock-outs, procurement delays, dependence on imported supplies (7, 30)	Disrupted testing services and inconsistent quality assurance
Financing	Chronic underfunding of laboratory services; high accreditation and surveillance costs (25, 30)	Limited implementation and sustainability of ISO 15189 programmes
Human resources	Brain drain, workforce shortages, inadequate competency-based training and limited ISO 15189:2022 awareness (7, 25)	Reduced institutional capacity to establish and sustain QMS
Organizational culture	Resistance to documentation, internal audits, occurrence reporting and SOP compliance (7, 20)	Weak quality culture and poor continuous quality improvement
Quality management systems	Fragmented, donor-driven implementation concentrated in disease-specific programmes (7, 25)	Limited facility-wide integration of QMS
Quality data and monitoring	Poor utilization of quality indicators, internal audits, management reviews and patient feedback (20, 25)	Weak evidence-informed quality improvement and risk management
Audit non-conformities	Persistent deficiencies in documentation, CAPA, occurrence management, quality indicators and internal audits (20, 25)	Delayed progression through SLIPTA and reduced readiness for ISO 15189 accreditation

### 3.4 Strategic recommendations for scaling up ISO 15189:2022 compliance in Nigeria

Scaling up ISO 15189:2022 implementation in Nigeria will require coordinated, system-wide interventions that address the regulatory, financial, technical, and workforce barriers identified in this review. Available evidence suggests that sustainable progress depends not only on strengthening individual laboratories but also on institutionalizing quality management within national health policies, financing mechanisms, and routine laboratory practice.

Regulatory and policy reforms should prioritize harmonizing the roles of key oversight institutions to eliminate jurisdictional overlap and establish a unified framework for laboratory quality assurance (7, 22, 32). Integrating ISO 15189:2022 into national health priorities, including HIV, tuberculosis, malaria, antimicrobial resistance surveillance, the Essential Diagnostics List, and Universal Health Coverage (UHC), would strengthen the role of laboratory accreditation as a core health system function rather than a stand-alone quality initiative (30, 43).

Strengthening institutional capacity remains equally important. Expanding competency-based education on quality management systems, risk management, internal auditing, and digital quality tools would equip laboratory personnel at all levels to implement the revised standard effectively. Existing quality improvement programmes such as SLMTA and SLIPTA provide practical, stepwise models that can serve as national platforms for progressive quality improvement while laboratories prepare for full ISO 15189 accreditation (24). These efforts should be complemented by mentorship networks linking accredited laboratories with facilities beginning their quality improvement journey, thereby facilitating peer learning and accelerating implementation (25, 44–46).

Long-term sustainability will depend on increased domestic investment in laboratory services. Financial support through dedicated government funding, targeted grants, subsidized accreditation costs, and strategic public-private partnerships would enable laboratories to strengthen infrastructure, adopt Laboratory Information Systems (LIS), expand external quality assessment programmes, and improve equipment maintenance (47–49). At the same time, strengthening workforce retention, succession planning, and continuous professional development will be essential to mitigate the impact of ongoing migration of skilled laboratory personnel.

Finally, implementation should be supported by standardized monitoring and accountability mechanisms. Routine internal audits, monitoring of quality indicators, periodic external assessments, and public recognition of accredited laboratories can reinforce continuous quality improvement while promoting transparency and public confidence in laboratory services (24, 50–52). Collectively, these interventions provide a practical framework for translating ISO 15189:2022 into sustained improvements in diagnostic quality and health system performance. The recommended actions, responsible stakeholders, implementation timelines, and suggested performance indicators are summarized in Table 5.

**Table 5:** Strategic recommendations for scaling up ISO 15189:2022 implementation in Nigeria.

Strategic priority	Key recommendations	Lead stakeholders	Suggested timeline	Key performance indicators (KPIs)
Strengthen governance and regulation	Harmonize regulatory mandates; establish a unified national laboratory quality framework; integrate ISO 15189 into national health policies (12, 30)	FMOH, MLSCN, NAFDAC, MDCN, NiNAS	Short term (1–2 years)	Harmonized regulatory framework; national implementation guideline adopted
Scale national quality improvement programmes	Expand SLMTA/SLIPTA implementation; establish phased national accreditation pathway; strengthen EQA participation (24, 48)	MLSCN, FMOH, NiNAS, ASLM	Short–Medium term (1–5 years)	Increased SLIPTA participation; progressive improvement in star ratings; increased number of ISO-accredited laboratories
Build workforce capacity	Expand competency-based QMS training; strengthen mentorship, internal auditor training and succession planning; improve staff retention (16, 24)	MLSCN, Universities, Professional Associations, FMOH	Continuous	Increased trained personnel; reduced workforce attrition; expanded mentorship programmes
Increase financing and infrastructure investment	Increase domestic funding; subsidize accreditation; strengthen PPPs; invest in LIS, equipment maintenance and EQA (7, 30)	Federal and State Governments, NHIA, Development Partners, Private Sector	Medium term (2–5 years)	Increased laboratory funding; improved infrastructure; wider LIS implementation
Promote digital transformation	Deploy LIS and electronic QMS; strengthen data management, document control and interoperability (12, 30)	FMOH, NCDC, NiNAS, ICT Partners	Medium term (2–5 years)	Increased LIS coverage; improved quality indicator reporting and audit readiness
Institutionalize monitoring and continuous improvement	Conduct routine internal audits; monitor quality indicators; strengthen management reviews; recognize high-performing laboratories (16, 48)	MLSCN, NiNAS, Laboratory Management	Continuous	Improved audit outcomes; increased accreditation success; sustained quality improvement

### 3.5 Case studies and best practices

Evidence from Nigeria and other sub-Saharan African countries demonstrates that successful implementation of ISO 15189 is achievable in resource-constrained settings when supported by sustained leadership, structured quality improvement programmes, and continuous mentorship. Across the reviewed studies, successful laboratories consistently adopted phased implementation strategies rather than attempting accreditation as a single event.

Within Nigeria, multiple implementation models demonstrate the feasibility of progressive quality improvement. Early experience from the Nigerian Institute of Medical Research (NIMR) established the importance of institutional leadership, documentation, and staff engagement in embedding quality systems (31), serving as a foundational baseline that catalyzed subsequent national tracking toward medical-specific frameworks. More recently, shifting the focus entirely to dedicated medical laboratory competence standards, the Nigeria Centre for Disease Control (NCDC) National Reference Laboratory and the Central Public Health Laboratory (CPHL) demonstrated how structured gap assessments, technical mentorship, SLMTA participation, and strong management commitment can facilitate progression from low SLIPTA ratings to validated ISO 15189 accreditation. Similarly, the Lagos State laboratory strengthening programme showed that coordinated government leadership, targeted investments, mentorship, and routine performance monitoring can substantially improve laboratory quality across multiple public facilities within a relatively short period (7).

Regional experiences reinforce these findings. Laboratories in Ghana, Kenya, and Malawi achieved measurable quality improvements through phased implementation, intensive mentorship, routine internal audits, and systematic monitoring of quality indicators despite operating under similar financial and infrastructural constraints (43, 53). A critical evaluation of these regional datasets reveals that absolute resource scarcity is not the definitive barrier to accreditation; rather, operational success depends heavily on centralized governance and programmatic consistency. Transition to ISO 15189:2022 further required strengthening risk-based thinking, method verification, patient-centred quality practices, and documentation systems, illustrating that compliance with the revised standard can be achieved through structured transition planning rather than wholesale infrastructural expansion.

Collectively, the reviewed evidence indicates that sustained leadership commitment, stepwise quality improvement programmes, technical mentorship, continuous staff capacity development, routine performance monitoring, and institutional ownership are the principal enablers of successful ISO 15189 implementation. These experiences provide practical models that can inform wider scale-up of ISO 15189:2022 across Nigeria (Table 6).

**Table 6:** Representative case studies and best practices for ISO 15189 implementation relevant to Nigeria.

Setting/Institution	Quality Improvement Strategy	Major Outcomes	Key Lessons for Nigeria
Nigerian Institute of Medical Research (NIMR), Nigeria (31)	Early QMS implementation; strong management commitment; structured documentation; staff engagement	Achieved ISO 9001:2008 certification and established one of Nigeria's earliest formal laboratory quality systems	Leadership commitment is fundamental for sustaining QMS implementation.
NCDC National Reference Laboratory & Central Public Health Laboratory (CPHL), Nigeria (33, 48)	Gap analysis; embedded technical advisers; SLMTA participation; development of quality manuals; corrective action training	Progressed from low SLIPTA ratings to 5-Star performance and ISO 15189 accreditation	Technical mentorship combined with institutional commitment accelerates accreditation readiness.
Lagos State Public Laboratory Improvement Programme, Nigeria (7)	Government-led intervention combining training, mentorship, equipment support and routine monitoring	Majority of participating laboratories improved SLIPTA ratings, with most attaining 3-Star status within one year	State ownership and structured mentorship can rapidly improve laboratory quality at scale.
Public Health Laboratory, Ghana (25, 44)	Phased accreditation; mentorship; section-by-section implementation; staff motivation	Achieved ISO 15189 accreditation despite resource limitations	Incremental implementation is more sustainable than attempting full accreditation simultaneously.
KEMRI HIV Laboratory, Kenya (49)	SLMTA-SLIPTA approach; intensive on-site mentorship; process improvement	External audit compliance improved from 47% to 94%, culminating in ISO 15189 accreditation	Continuous mentorship and performance monitoring produce substantial quality gains.
Malawi-Liverpool-Wellcome (MLW) Research Programme, Malawi (45)	ISO 15189:2022 transition through risk-based planning, method verification, quality indicators and revised documentation	Successfully aligned laboratory operations with ISO 15189:2022 requirements	Transition to ISO 15189:2022 requires strengthening risk management, verification procedures and patient-centred quality systems rather than major infrastructure alone.

### 3.6 Impact of successful implementation of ISO 15189:2022 standards

Successful implementation of ISO 15189:2022 has consistently been associated with improvements in diagnostic quality, patient safety, laboratory efficiency, workforce competence, and health system performance. Evidence synthesized from Nigeria and comparable low- and middle-income settings indicates that accreditation extends beyond compliance, serving as a mechanism for strengthening clinical governance and health system resilience (Table 7).

**Table 7:** Reported impacts of ISO 15189 implementation relevant to Nigeria.

Impact Domain	Evidence from Literature	Relevance to Nigeria
Diagnostic quality	Reduced analytical errors, improved test accuracy, lower contamination rates, and greater compliance with international quality standards (3, 47)	Improves reliability of laboratory diagnosis and clinical decision-making.
Patient safety	Fewer diagnostic errors, more appropriate treatment decisions, improved clinical outcomes, and increased patient confidence (54)	Enhances patient safety and quality of care across all levels of the health system.
Operational performance	Reduced specimen rejection, shorter turnaround times, fewer client complaints, improved workflow efficiency, and stronger quality indicator monitoring (47, 53)	Improves laboratory productivity, efficiency, and service delivery.
Workforce capacity	Continuous competency assessment, structured training, stronger quality culture, and improved staff performance (49, 55)	Builds a competent workforce capable of sustaining accreditation.
Institutional credibility	Increased confidence among clinicians, patients, regulators, and development partners; greater opportunities for collaboration and investment (22, 49)	Strengthens public trust and enhances institutional reputation.
Health system strengthening	Improved disease surveillance, outbreak preparedness, antimicrobial stewardship, internationally recognized laboratory results, and stronger health security (3, 22)	Supports UHC, public health emergency preparedness, and long-term health system resilience in Nigeria.
Economic impact	Improved confidence in domestic diagnostic services may reduce dependence on overseas healthcare and associated medical tourism (3, 47)	Potentially lowers healthcare expenditure leakage while increasing returns on national laboratory investments.

Implementation of ISO 15189-based quality management systems improves analytical accuracy by reducing laboratory errors, specimen rejection, and contamination rates while enhancing conformity with international quality standards (47). These improvements support more reliable diagnoses and appropriate clinical decision-making, particularly in high-burden settings where diagnostic accuracy directly influences patient outcomes. Studies also demonstrate improvements in disease detection following implementation of structured quality systems, including increased diagnostic sensitivity in tuberculosis reference laboratories.

Patient safety benefits primarily through reductions in diagnostic errors and greater consistency of laboratory processes, minimizing inappropriate treatment and improving confidence in laboratory results (47, 54). Accreditation additionally strengthens laboratory credibility among clinicians, patients, regulators, and development partners, improving institutional reputation and facilitating national and international collaborations (49).

At the operational level, accredited laboratories consistently report improved turnaround times, fewer service complaints, lower specimen rejection rates, and more efficient workflow management (49, 53). Continuous competency assessment and staff development further strengthen workforce performance and promote a sustained culture of quality improvement (47, 55).

Beyond individual laboratories, wider implementation of ISO 15189:2022 has important health system implications. Reliable diagnostic services strengthen disease surveillance, outbreak preparedness, antimicrobial stewardship, and public confidence in healthcare services (3, 22, 49). For Nigeria, expanding technical competence and accreditation capacity could also reduce dependence on overseas diagnostic services and medical tourism while supporting Universal Health Coverage (UHC) and broader national health security objectives.

### 3.7 Strengths and limitations of the review

This review has several strengths. It employed a structured narrative review approach with transparent reporting guided by the Scale for the Assessment of Narrative Review Articles (SANRA) and a PRISMA-informed study selection process. By extending the evidence synthesis through July 2026, it provides one of the earliest comprehensive assessments of laboratory quality management in Nigeria following the completion of the global transition to ISO 15189:2022 in December 2025. The review further integrates the Clinical and Laboratory Standards Institute (CLSI) Quality System Essentials framework with the WHO-AFRO Stepwise Laboratory Quality Improvement Process Towards Accreditation (SLIPTA) checklist, enabling a systems-level evaluation of governance, accreditation, workforce, infrastructure, quality improvement programmes, and implementation experiences. The inclusion of representative Nigerian and regional case studies also strengthens the practical relevance of the recommendations for policy and practice.

The findings should nevertheless be interpreted in light of certain limitations. As a structured narrative review, the study provides qualitative synthesis rather than quantitative meta-analysis and therefore cannot estimate pooled effect sizes or causal relationships. Although a systematic search strategy, supplementary hand searching, and predefined eligibility criteria were used to minimize bias, the review remains susceptible to selection and publication biases, particularly because unsuccessful laboratory quality improvement initiatives and lower-performing laboratories are less frequently reported in the published literature. In addition, no formal risk-of-bias appraisal of individual studies was undertaken, consistent with the narrative review design. Finally, because the transition to ISO 15189:2022 was only completed globally in December 2025, evidence on long-term implementation outcomes and national adoption under the revised standard remains limited and continues to evolve.

## 4 Conclusion

This review demonstrates that ISO 15189:2022 provides a comprehensive framework for strengthening laboratory quality management and accreditation in Nigeria. Although important progress has been achieved through national regulatory structures, stepwise quality improvement programmes, and successful accreditation of selected laboratories, implementation remains limited by regulatory fragmentation, inadequate financing, infrastructure deficits, workforce shortages, and weak institutional capacity. Scaling up structured mentorship, competency-based training, digital quality systems, and sustained government investment will be critical to accelerating nationwide adoption. Future research should evaluate cost-effective implementation models, long-term sustainability, digital innovations, and workforce retention strategies to inform policy and practice. Ultimately, successful implementation of ISO 15189:2022 has the potential to strengthen diagnostic quality, improve patient safety, and build a more resilient and equitable healthcare system in Nigeria.
